# Imaging Large Cohorts of Single Ion Channels and Their Activity

**DOI:** 10.3389/fendo.2013.00114

**Published:** 2013-09-04

**Authors:** Katia Hiersemenzel, Euan R. Brown, Rory R. Duncan

**Affiliations:** ^1^Edinburgh Super-Resolution Imaging Consortium (ESRIC), Institute of Biological Chemistry, Biophysics and Bioengineering, School of Engineering and Physical Sciences, Heriot-Watt University, Edinburgh, UK

**Keywords:** PALM, storm, STED, super-resolution, TIRFM, imaging, microscopy, ion channel

## Abstract

As calcium is the most important signaling molecule in neurons and secretory cells, amongst many other cell types, it follows that an understanding of calcium channels and their regulation of exocytosis is of vital importance. Calcium imaging using calcium dyes such as Fluo3, or FRET-based dyes that have been used widely has provided invaluable information, which combined with modeling has estimated the subtypes of channels responsible for triggering the exocytotic machinery as well as inferences about the relative distances away from vesicle fusion sites these molecules adopt. Importantly, new super-resolution microscopy techniques, combined with novel Ca^2+^ indicators and imaginative imaging approaches can now define directly the nano-scale locations of very large cohorts of single channel molecules in relation to single vesicles. With combinations of these techniques the activity of individual channels can be visualized and quantified using novel Ca^2+^ indicators. Fluorescently labeled specific channel toxins can also be used to localize endogenous assembled channel tetramers. Fluorescence lifetime imaging microscopy and other single-photon-resolution spectroscopic approaches offer the possibility to quantify protein–protein interactions between populations of channels and the SNARE protein machinery for the first time. Together with simultaneous electrophysiology, this battery of quantitative imaging techniques has the potential to provide unprecedented detail describing the locations, dynamic behaviors, interactions, and conductance activities of many thousands of channel molecules and vesicles in living cells.

## Exocytosis

Ion channel biology is central to all physiology and regulated exocytosis, the process of secretion in specialized cells such as neurons and neuroendocrine cells, underlies physiological cell-to-cell signaling (1). Mis-regulation of exocytosis leads variously to a number of different conditions, including diabetes (2), neurological disorders (3), growth and sleep disorders (4), and asthma (5). Regulated exocytosis does not proceed spontaneously, but requires an influx of calcium ions through voltage-gated calcium channels (VGCCs), with the final fusion event at the plasma membrane driven by synaptotagmin-dependent mechanotransduction (6, 7) to the core SNARE complex comprising syntaxins, SNAP-25/23/29 (both target, or t-SNAREs) and synaptobrevins (a vesicular, or v-SNARE) (8–10) as well as SM proteins, such as munc18 (11–14). In both neurons and endocrine cells the voltage-dependent calcium influx is typically shaped by a classical negative feedback role for large-conductance calcium- and voltage-activated potassium (BK) channels (15–17). Thus disruption of either VGCC and/or BK channel function also leads to defects in physiology (18–20) [for example, chronic inflammatory pain is associated (21) with exon-18 splice variants (22) that lack the putative motif (“synprint”) (23) in N-type VGCCs that is thought to interact directly with the secretory machinery]. Central to all these models is the requirement for secretory vesicles to be appropriately localized with the secretory machinery and VGCC and BK channels. Our understanding of ion channel biology has been defined largely by electrophysiological approaches, due to their high temporal resolution and single molecule sensitivity. Recent, rapid advancements in super-resolution imaging now offer the opportunity to test directly long-standing hypotheses regarding ion channel locations, interactions, dynamics, and compositions in living cells.

## Voltage-Gated Calcium Channels and Exocytosis

Voltage-gated calcium channels are the voltage sensors that convert cell depolarization into a cytoplasmic calcium signal, which subsequently triggers regulated exocytosis. A multiplicity of calcium channel subtypes is expressed in excitable cells (including neurons, chromaffin, and pancreatic beta cells as excitable secretory cell types) (24–26). Seven genes encode high voltage-activated channels (HVA) L (Ca_v_ 1.1–1.4), P/Q (Ca_v_ 2.1), N (Ca_v_ 2.2), and R (Ca_v_ 2.3) type. Three genes encode low voltage-activated channels (LVA, Ca_v_ 3.1–3, known as T-type channels). In neurons and endocrine cells VGCCs regulate a variety of other fundamental cellular processes in addition to controlling vesicle fusion; decades of research have defined electrophysiologically the relative contribution of each subtype to secretion (19, 25). For example, in mouse adrenal chromaffin cells, responsible for catecholamine (e.g., adrenaline) secretion, and in pancreatic beta cells, which release insulin in response to elevated blood glucose, N- and L-type calcium channels play a major role in both exocytosis and in other cellular signaling such as shaping the action potential and pacemaker currents in chromaffin cells but N-, P/Q-, and R VGCCs are also present. Importantly, our understanding of the spatial organization of the different VGCC subtypes is immature (Table 1) and in this respect their interactions and spatio-temporal patterning in the functional- and molecular-vicinity of the fusion complex is of particular interest.

**Table 1:** **Voltage-gated calcium channel cohort activities that have been imaged**.

Channel type	System	Optical calcium events term	Imaging technique	Conditions	Reference
L-type	Rat myocytes	Sparkletts	Confocal	Enhanced extracellular Ca^2+^ (20 mM) required for imaging	(27, 28)
	Rat myocytes	Sparkletts	TIFRM using Fluo-f5	Calcium channels in clusters	
T-type	nd	nd	nd	Nd	nd
N-type	*Xenopus* oocytes	Single channel calcium fluorescence transients (SCCaFTs)	TIFRM using fluorescent calcium indicator (Fluo-4)	Heterologously expressed channels	(29)
P/Q type	nd	nd	nd	Nd	nd

*nd = not done*.

## Large-Conductance Calcium Activated Potassium Channels and Exocytosis

As BK channels, encoded by a single gene, have a key role in modulating the calcium signal that leads to vesicle fusion (18–20) there has been considerable effort in trying to estimate the distances between Ca^2+^ and K^+^ channels, their inter-channel interactions (physical and functional) (16, 30, 63), VGCC and BK interactions with the exocytotic machinery itself, particularly with syntaxin1 (21, 31–33), and in the distances between these channels and large dense-cored vesicles (64). Mathematical modeling of calcium concentration nano-domains near the intracellular mouth of channels, combined with the determination of the Ca^2+^ sensitivity of the secretory machinery (i.e., synaptotagmin) have led to the concept that secretory vesicles reside very close to VGCCs in the membrane, and slightly further away from BK channels (34–37, 64). Determining the precise concentration of Ca^2+^ required to elicit exocytosis is confounded by spatial heterogeneity in the cell that to date has been impossible to measure accurately; however, it is likely that fusion competent vesicles in secretory cells are targeted somehow to the sites at the membrane experiencing high Ca^2+^ concentrations – i.e., very close to the mouth of a calcium channel (34).

## Functional Coupling of Ion Channels with Exocytosis

Our understanding of the functional coupling of ion channels with the secretory apparatus comes from diffraction-limited microscopy, binary biochemical binding determined *in vitro*, and electrophysiology. The first can only resolve, at the very best, clusters of ion channels and approximate distances in images in the order of 250 nm. *In vitro* biochemistry, whilst invaluable, cannot deliver the “where’s and when’s” of interactions in cells and so overlooks the key elements of spatio-temporal regulation. Electrophysiology can resolve single ion channel activities, or entire cell cohorts of activity, but with limited spatial resolution. It thus remains unknown how: (i) membrane cohorts of single ion channels are spatially distributed, (ii) the proportion of *active* ion channels compared to the total pool (Ca^2+^ and K^+^) reside within functionally meaningful distances of fusion competent- and/or incompetent-vesicles, (iii) how the dynamics of channel activity may correlate with their spatial pattern *and/or* interactions with the SNARE molecular machinery in intact cells, and (iv) whether every channel at the membrane is functional. It is clear that new tools are required to address these questions.

## Super-Resolution Imaging and Exocytosis

The membrane-trafficking field has a strong history of using cutting-edge techniques and imaging is no exception. Super-resolution microscopy is an emerging powerful tool to further research on ion channels and calcium signaling involved in exocytosis, and have already been applied in studies of the exocytotic machinery (12, 38–41). Our own recent work revealed that the majority of vesicles do not access the necessary compliment of SNARE molecular machinery at the membrane required for fusion (12, 40, 42). Furthermore, vesicle dynamics are also segregated, not only spatially at the membrane but also by vesicle age as we showed that vesicles are prioritized for release according to the time since their assembly (43). This mini-review summarizes the main super-resolution imaging modalities and illustrates their potential uses in quantifying ion channel molecular biology in relation to exocytosis. Table 2 summarizes the super-resolution imaging modalities described and their (potential) uses in examining ion channel biology.

**Table 2:** **Summary of available super-resolution microscopy and spectroscopic approaches and their potential for ion channel imaging**.

Imaging modality	Description and potential for ion channel imaging	Reference
STED	Genuine sub-diffraction-limit imaging using a “depletion” laser to reduce the size of the point-spread-function. Resolution to ∼50 nm, potential for resolving small channel clusters at the plasma membrane	(49–53)
TIRFM	Limits the excitation in a sample to a thin (100 s of nanometers) optical section primarily at the base of a cell adhered to a glass cover-glass. The high contrast and rapid imaging data delivered makes this approach ideal for examining ion channel distributions, trafficking, and movements at the cell surface, with diffraction-limited resolution. Used for optical patching to localize ion channel activity	(13, 41–43)
SIM	Illumination of the sample with a known pattern allows the mathematical reconstruction of images from moiré fringes, thus revealing high-frequency, sub-diffraction structures. Potential for visualizing ion channel clusters (resolution ∼85 nm) or intracellular trafficking	(58, 59)
PALM	Localization microscopy that determines the location of single molecule fluorescent signals. Separates signals in time by photo-activating subsets of fluorescent proteins repetitively. Ideal for quantifying the spatial arrangements of cohorts of single channel subunits	(44, 45)
STORM/GSDIM/DSTORM	Localization microscopies that determine single molecule locations. Separates signals in time by photo-switching subsets of fluorescent molecules from bright to dark, or spectral forms. May be used with immunodetection to localize cohorts of endogenous channel subunits with 5–20 nm certainty	(42, 46)
sptPALM	Single-particle-tracking PALM, localizes photo-activated fluorescent proteins in living cells over time to allow the tracking of single molecules. Ideal for quantifying the movements of cohorts of single ion channels at the cell surface with 20–50 nm certainty	(12, 36, 48)
Fluorescence lifetime imaging microscopy (FLIM)	Quantifies the fluorescence lifetime of a fluorophore to aid with either contrast (by measuring an additional parameter in an image dataset) or in particular, to quantify FRET. Ideal for quantifying ion channel molecular interactions anywhere in 3-D in a cell	(20, 21, 42–47)
Fluorescence correlation spectroscopy (FCS)	Quantifies the diffusion of single fluorescent molecules through small excitation volumes in 3-D. Delivers directly molecular number, concentration, diffusion rates, and potentially interactions from living samples or using purified samples *in vitro*. Ideal for quantifying dynamic ion channel molecular behaviors	(56–59)

## Total Internal Reflection Fluorescence Microscopy

Total internal reflection fluorescence microscopy (TIRFM) is an optical technique that allows the evanescent illumination of a thin optical section (∼100 nm) near the base of a cell (44). For TIRFM the use of the high NA objective becomes relevant in combination with the low refractive index of aqueous cell media. When the illumination laser encounters the interface between these refractive indices at a shallower angle than the so-called critical angle, then total internal reflection occurs, creating an evanescent wave at the interface. This extends and illuminates with an exponential decay only about 100 nm into the specimen before it becomes too weak to excite fluorophores. This creates a high-contrast image of the plasma membrane and adjacent structures with little background fluorescence from the intracellular compartments of the cell. This allows the imaging of ion channels, vesicles, and exocytotic proteins in living samples with high temporal resolution (13, 42, 45, 46). TIRFM is the foundation for a number of super-resolution approaches that take advantage of the thin optical section generated in order to reduce out-of-focus signal.

## Single Molecule Localization Microscopies

**Figure 1:**
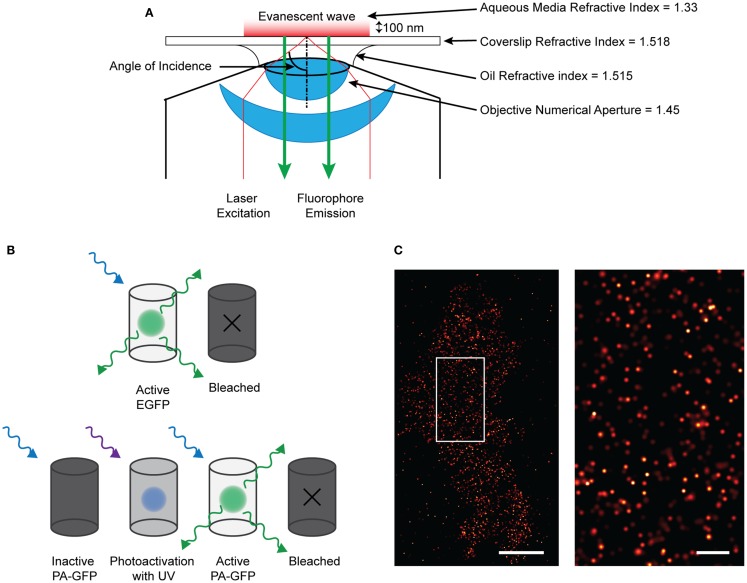
**Single molecule imaging using PALM technique. (A)** Diagram of the TIRF microscopy setup. By angling the excitation light beam though a combination of the refractive indices of the lense, oil, coverslip, and aqueous media, the beam is reflected at the glass/media interface and is directed back into the objective. This creates an evanescent wave at the interface which effectively excites fluorophores only about 100 nm into the sample. Therefore only those fluorescent proteins or dyes within that region will be excited and emit fluorescence which can be detected. **(B)** Demonstrating the difference between EGFP and PA-GFP. EGFP will absorb and emit light without prior activation before bleaching off. PA-GFP needs to be activated by short-wavelength light before it is confirmationally able to absorb excitation and emit light. **(C)** (left) Localized and rendered PALM image of BK channels on the plasma membrane of a HEK293 cell (scale bar: 5 μm) with a region of interest (right) showing the localization of molecules in detail (scale bar: 1 μm).

An impressive pallet of fluorescent proteins (FP) has been created, each with different excitation and emission wavelengths. Not only has the color range been refined, but also the use: through modification of the molecular structure, photo-activatable (PA) FPs have been developed, permitting applications in advanced microscopy such as photoactivation localization microscopy (PALM) (47, 48). Techniques such as PALM, ground-state depletion with immediate molecular return (GSDIM) (49), and stochastic optical switching microscopy (STORM) (50) have circumvented the diffraction-limit of resolution to permit the localization of single molecule point-spread-function (PSF) signals that are separated in time. STORM and GSDIM detect PSF-signals from single organic dye molecules; a potentially high density of molecules (e.g., fluorescent-conjugated antibody-detected epitopes) in the sample is driven into a long-lived, optically inactive “dark state” using a combination of high illumination power and specialized, highly reducing buffers. Single fluorescent molecules then spontaneously and randomly re-emerge to emit photons during subsequent imaging (Figure 1). This permits the acquisition of single PSF-signals that are sparsely distributed in a time-resolved dataset. PALM employs PA encodable FP (47, 48). These molecules are inherently non-fluorescent but can be altered conformationally, using short-wavelength illumination in order to render them fluorescent. By determining empirically the dose of activation energy required in order to activate a sparse fraction of all PA-molecules, again, single PSF-signals can be acquired. Rapid imaging trains capture the fluorescent data, before single molecules are irreversibly photo-destroyed by bleaching and other processes. Cycles of activation, followed by imaging and photo-destruction are repeated over and over again acquiring 1000 s of images in total. PALM is already starting to be used to define ion channel molecule localization, recently employed to image single aquaporin molecules (51).

Single molecule signals can be identified by their shape, size, expected fluorescent intensity (i.e., they are PSFs) as well as by their quantal “on–off” behavior. Mathematical fitting, localizing the centroid of these PSFs defines the precise *xy* coordinates from where the signals arose; the certainty of localization is affected by brightness, noise, and pixel size. By rendering coordinates, a virtual image is created which shows the coordinate positions of all the molecules on the plasma membrane that have emitted. A variation of PALM combines single-particle tracking, so termed sptPALM (52), allowing the high-precision tracking of many 1000 s of single molecule signals with high temporal resolution.

## Simulated Emission Depletion Microscopy

**Figure 2:**
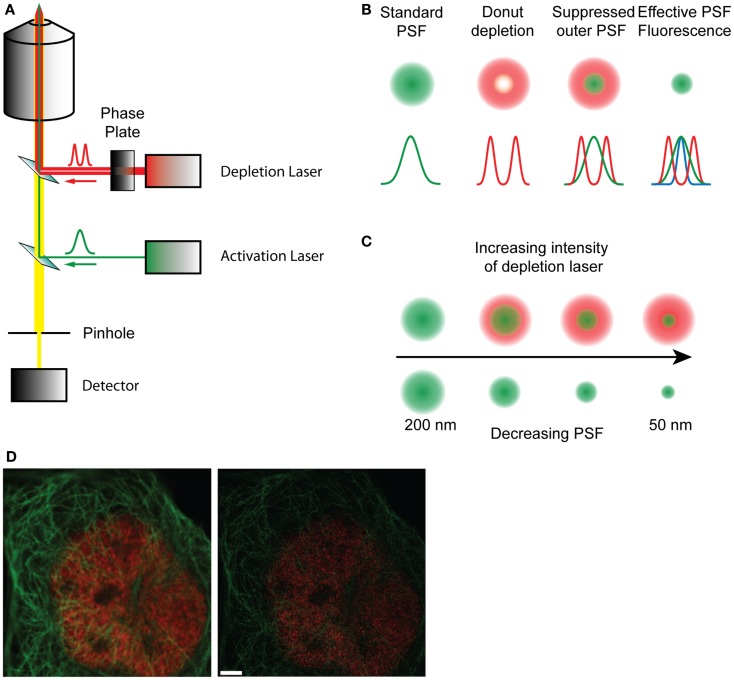
**(A)** Schematic diagram of a STED microscope setup. The activation laser is overlaid with the depletion laser that passes through a phase plate, and focused to a focal point in the sample. The fluorescence emitted by the sample passes through a pinhole and is collected in the detector. **(B)** Representation of how the effective point spread function is formed by the overlap of the standard PSF and the depletion PSF. **(C)** With increasing intensity of the depletion laser the shape of the donut changes, decreasing the size of the inner ring and therefore reducing the effective PSF of the fluorophore from 200 nm to about 50 nm, effectively increasing resolution. **(D)** A raw confocal (left) and STED image (right) showing the improvement in detail and resolution. Red stain – nuclear pores, green stain – microtubules. Scale bar, 500 nm.

The underlying limitation with microscopy is the diffraction-limited width of the PSF. Single molecule localization microscopies (SMLMs) circumvent this by determining the centroids derived from single molecule PSFs, and though these are excellent techniques, they are not imaging directly sub-diffraction structures. Simulated emission depletion microscopy (STED) presents a fundamentally different approach, directly manipulating the PSF through the use of stimulated emission to deplete fluorophores before they fluoresce (53). On a conventional scanning microscope, the sample is raster scanned with an excitation beam, which increases the energy of the fluorophores from the ground state *S*_0_ to the exited singlet state *S*_1_. The fluorophores spontaneously relax, emitting photons as they return to the ground state, which is detected as fluorescence. This process typically occurs on a nanosecond timescale. In STED microscopy a second depletion laser, is used immediately after the excitation beam (Figure 2). The depletion laser forces the excited fluorophores back to the ground state via stimulated emission, a process that typically occurs on a picosecond timescale. This effectively forces depleted fluorophores into a dark state before traditional spontaneous fluorescence can occur. By using a phase mask profile with a donut-shaped depletion pattern, that perfectly overlaps the initial excitation beam, the effective PSF becomes defined by the geometry of the donut inner ring and the depletion laser power. The optical resolution can be considerably improved, to around 70 nm, to give a higher resolution data compared to standard diffraction-limited image. Gated-STED (gSTED) (54) combines this with time-correlated detection and pulsed excitation to further increase resolution, reduce noise, and improve utility with living samples enormously by reducing illumination energies (54–56). STED has been used to examine Ca^2+^ channel clustering induced by the exocytotic machinery (57) as well as probing the nano-scale structure of the synapse (38, 39, 58). The advent of less cytotoxic gSTED technologies will increase the utility of this approach in cell biology.

## Structured Illumination Microscopy

Structured illumination microscopy (SIM) illuminates samples with a grid pattern and a sinusoidal excitation, providing a known high-frequency structured pattern (59, 60). Mathematical approaches are then applied to reconstruct a real image from the emission data, with the ability to resolve structures at around half the diffraction-limit (i.e., around 85 nm resolution in general). Importantly, SIM also improves the axial resolution in images and can be used anywhere in 3-D, albeit in around 300 nm *z*-volumes, in multiple spectral colors. SIM has not yet been used to examine ion channel biology, to our knowledge, but clear opportunities exist, perhaps for studying intracellular channels beyond the reach of TIRF-limited approaches.

## Quantifying Protein–Protein Interactions in Cells

Förster resonance energy transfer (FRET) is a physical effect where energy can be transferred in a non-radiative way between a high-energy fluorescent donor molecule and a lower energy proximal acceptor, if the absorption and emission spectra of each, respectively, overlap. As FRET is strongly dependent on the inter-fluorophore distance and falls off with the sixth-power of distance, it occurs only on the nanometer scale. For this reason, FRET between two fluorescent molecules is commonly interpreted as indicating a direct interaction (61). Quantifying FRET is notoriously unreliable however, especially when using fluorescence intensity as a read-out. When FRET occurs, the energy transfer from the donor to an acceptor provides a rapid route to relaxation for the excited donor molecule, resulting in a significantly shortened fluorescence lifetime. Measuring the excited-state fluorescence lifetime of the donor molecule in each pixel of an image, known as fluorescence lifetime imaging microscopy (FLIM), therefore provides a direct and quantitative approach to this problem. FLIM has been widely used to quantify FRET between the SNAREs and binding partners inside intact cells (20, 21, 42–46, 48) and could be applied similarly to the study of ion channels; for example, it has been long-thought that VGCCs containing the so-called “synprint” (31) site interact directly with syntaxin and synaptotagmin but this has never been measured directly in intact cells.

## Fluorescence Correlation Spectroscopy

Although powerful, FLIM suffers from slow temporal resolution, as commonly 10,000–100,000 s of photons must be acquired from each pixel in the image in order to build a large enough sample of time-tagged photons to permit accurate data fitting (62). In addition, in FRET experiments, the absence of a detected FRET signal does not necessarily mean that no interaction has occurred, leading to potential false-negative conclusions. Fluorescence Correlation Spectroscopy (FCS) acquires intensity fluctuations caused by fluorescence emission as molecules diffuse across a tiny excitation volume held stationary in a living sample (63–65). This approach thus calculates the diffusion rates, concentrations, and molecular numbers of molecules under scrutiny. As diffusion depends directly upon mass, a simultaneous decrease in diffusion rate and cross-correlation of signals between two different fluorescent molecules in the excitation volume provides unequivocal evidence of an interaction, on the millisecond timescale.

## Super-Resolution Imaging of Ion Channels

Single molecule localization microscopy can be applied to ion channels (51), to define the precise locations of large cohorts of single molecules. Furthermore, it is possible to combines SMLMs with diffraction-limited imaging, in order to capture data describing the locations of single channels and single vesicles, for example. This approach would allow appraisals such as “nearest-neighbor” analyses, to determine the distances between vesicle centers and their adjacent ion channel molecules. These approaches were used recently, finding that in contrast to previous understanding, the majority of the secretory machinery is segregated spatially from secretory vesicles, with protein molecules moving between membrane “hotspots” distinct from vesicle docking sites (12, 40).

## Imaging Ion Channel Activity – “Optical Patching”

Patch-clamp electrophysiology is the ideal technique to determine the functionality of single ion channel molecules. Combined with fluorescent Ca^2+^ indicators, voltage-clamping can be used to deliver a train of tiny depolarizations, designed to open and close ion channels very rapidly. Image processing approaches may then be used, similarly to SMLM approaches, to localize the centroid position of the fluorescent signal reporting the ion channel location in a technique called optical patching (66–68). This approach has been used to great effect to report the locations of large-conductance, long open-dwell time IP3 receptors in cells (66); with the advent of image data “de-noising” strategies (40, 69) and faster camera detectors, optical patching promises to be useful for localizing single VGCCs in living cell membranes, combined with TIRFM.

## Imaging Ion Channel Tetramers

Active VGCCs and K channel alpha subunits are known to be tetramers; it difficult, however, to determine whether the channels detected on the cell surface using any of the approaches described above, are assembled alpha subunits. The study of ion channel function has been aided greatly over the years by the discovery that several potent natural toxins bind to specific ion channel targets with exquisite sensitivity and affinity. Recent work has taken advantage of this, using fluorescent-conjugates of these toxins to localize ion channels in intact cells. For example, ω-conotoxin is a peptidyl toxin originally isolated from the fish-hunting cone snail *Conus magnus* (70, 71) and is a specific N-type calcium channel blocker. This toxin has been used in the past to label neurons and neuromuscular junctions, revealing ion channel localization in diffraction-limited images (72). These approaches can now be combined with SMLM, as recently described using a Na^+^-channel toxin in order to define the nano-scale locations of single assembled tetrameric alpha subunits (73). Combined with patch-clamp electrophysiology, these approaches offer the promise of manipulating channel behavior and localizing large cohorts of single molecules, simultaneously.

In summary, ion channel biology is uniquely placed in having diverse single molecule-resolution biophysical approaches available; electrophysiology and the newly emerging super-resolution imaging and spectroscopic approaches. Together, these promise to further our understanding not only of ion channel behavior, but also of channel location, function, and relationship with vesicles and the exocytotic molecular machinery.

## Conflict of Interest Statement

The authors declare that the research was conducted in the absence of any commercial or financial relationships that could be construed as a potential conflict of interest.
